# Supplementary material for: Limited Recognition of Highly Conserved Regions of SARS-CoV-2
Source: Microbiol Spectr. 2022 Feb 23;10(1):e02780-21. doi: 10.1128/spectrum.02780-21 (PMC8865427; doi:10.1128/spectrum.02780-21)
Supplement: SUPPLEMENTAL FILE 1 — Supplemental material. Download SPECTRUM02780-21_Supp_1_seq1.pdf, PDF file, 0.4 MB [file spectrum02780-21_supp_1_seq1.pdf]

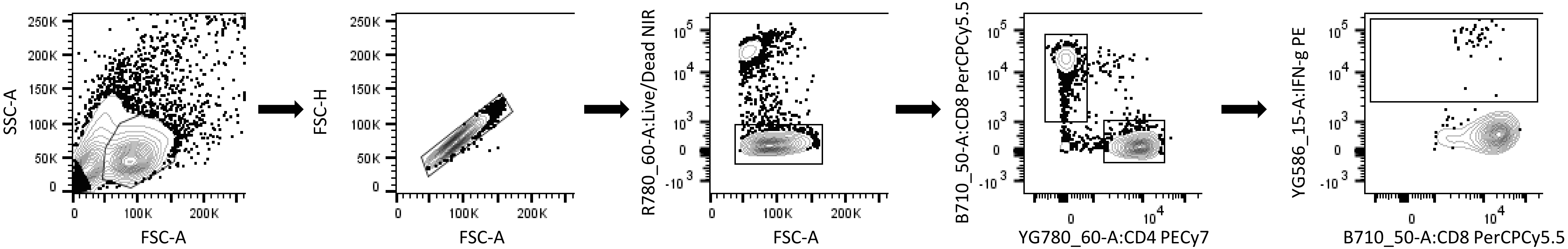

Supplementary Figure 1: Gating strategy used to detect SARS-CoV-2 specific IFN-γ producing CD8+ T cells. Lymphocytes were gated using FSC-A vs SSC-A, then doublets excluded using FSC-A vs FSC-H. Viable cells were isolated using Live-Dead NIR, CD8+CD4- cells were then gated and IFN-γ production determined.

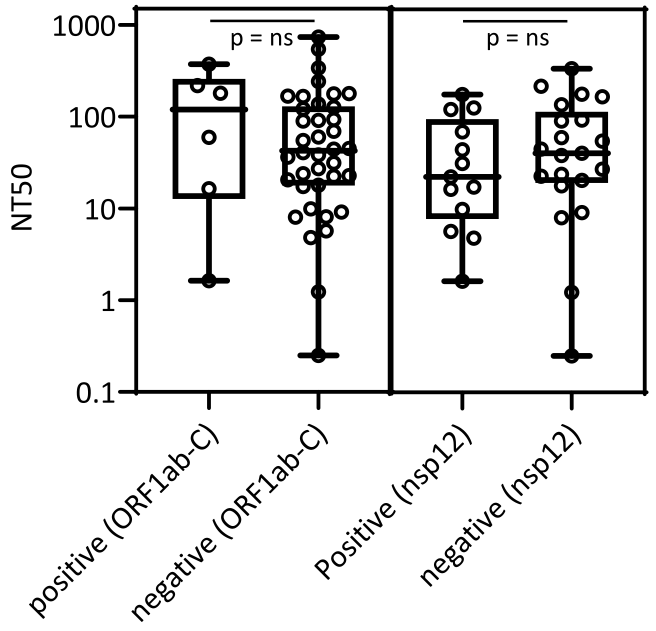

Supplementary Figure 2: Partipcants were stratified based upon the presence or absence of CD8^{+} T cell responses directed against the different SARS-CoV-2 and their neutralising titre 50 (NT50) compared. Statistical analysis was performed in GraphPad Prism using an unpaired Mann-Whitney Test. Differences were considered statistically significant if $p<0.05$.

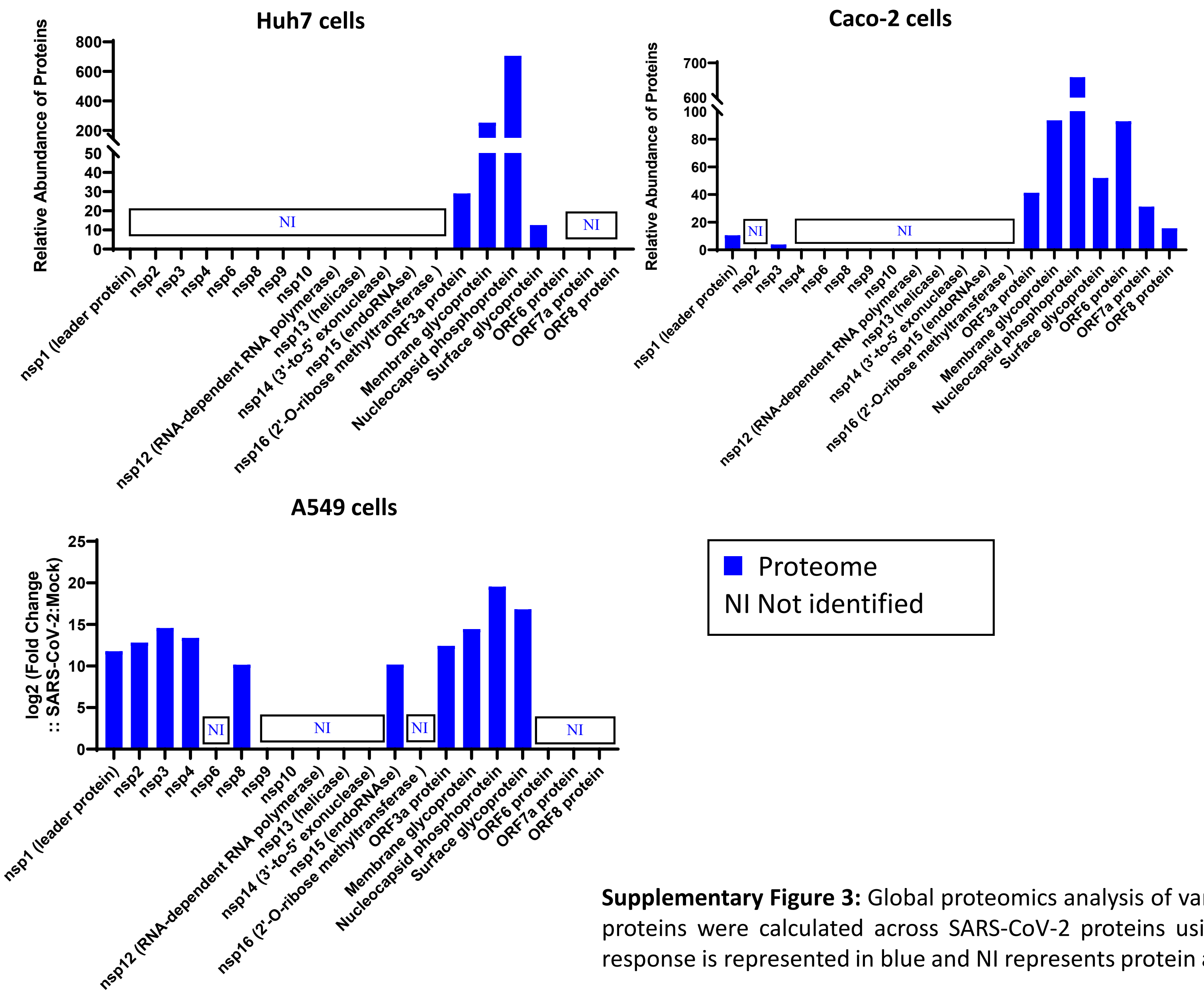

Supplementary Figure 3: Global proteomics analysis of various cells infected with SARS-CoV-2 isolates. Relative abundance of proteins were calculated across SARS-CoV-2 proteins using Huh7, Caco-2 and A549 cells. The relative protein abundance response is represented in blue and NI represents protein abundance not identified across global proteomic analysis.

Supplementary Table 1: Predicted strong binding epitopes encoded by the conserved orf1ab peptides

Position	HLA	Peptide*	Score	Aff(nM)
3009	HLA-A*23:01	DYYRSLPGVF	0.5808	93.2
3009	HLA-A*24:02	DYYRSLPGVF	0.5105	199.7
3010	HLA-A*23:01	YYRSLPGVF	0.5449	137.6
3010	HLA-A*24:02	YYRSLPGVF	0.529	163.5
3010	HLA-C*04:01	YYRSLPGVF	0.1995	5771.6
3010	HLA-C*06:02	YYRSLPGVF	0.4186	539.7
3010	HLA-C*07:02	YYRSLPGVF	0.6517	43.3
3011	HLA-C*06:02	YRSLPGVF	0.3341	1346.2
3011	HLA-C*07:02	YRSLPGVF	0.3935	707.8
3012	HLA-C*15:02	RSLPGVFCGV	0.4442	409.1
3013	HLA-A*02:01	SLPGVFCGV	0.7866	10.1
3014	HLA-B*51:01	LPGVFCGV	0.2777	2477.9
4554	HLA-C*04:01	WYDFVENPDI	0.238	3808.2
4554	HLA-C*04:01	WYDFVENPDIL	0.2571	3095.8
4718	HLA-B*51:01	FGPLVRKIFV	0.2746	2563.6
4722	HLA-C*06:02	VRKIFVDGV	0.3488	1148.4
4724	HLA-A*02:01	KIFVDGVPFV	0.8011	8.6
4725	HLA-C*04:01	IFVDGVPFV	0.1987	5822.2
4725	HLA-C*04:01	IFVDGVPFVV	0.214	4935
4726	HLA-A*01:01	FVDGVPFVV	0.3954	693.4
4726	HLA-A*02:01	FVDGVPFVV	0.772	11.8
4726	HLA-C*01:02	FVDGVPFVV	0.2349	3937.3
4726	HLA-C*04:01	FVDGVPFVV	0.262	2935.5
4726	HLA-C*15:02	FVDGVPFVV	0.6091	68.7
4730	HLA-B*35:01	VPFVVSTGY	0.7371	17.2
4731	HLA-A*23:01	PFVVSTGYHF	0.5897	84.8
4731	HLA-A*24:02	PFVVSTGYHF	0.4971	230.7
4732	HLA-B*35:01	FVVSTGYHF	0.6778	32.7
4732	HLA-C*03:04	FVVSTGYHF	0.5566	121.2
4800	HLA-A*11:01	QTVKPGNFNK	0.649	44.6
4801	HLA-A*11:01	TVKPGNFNK	0.69	28.6
4926	HLA-C*03:04	NVIPTITQM	0.5836	90.5
4928	HLA-B*07:02	IPTITQMNL	0.5407	144
4928	HLA-B*51:01	IPTITQMNL	0.3128	1695
4928	HLA-A*11:01	IPTITQMNLK	0.6309	54.2
4933	HLA-B*08:01	QMNLKYAI	0.5936	81.2
4934	HLA-A*03:01	MNLKYAISAK	0.6612	39.1
4944	HLA-C*06:02	NRARTVAGV	0.3196	1574.1
5005	HLA-A*03:01	HLMGWDYPK	0.6794	32.1

5005	HLA-A*11:01	HLMGWDYPK	0.7572	13.8
5011	HLA-B*07:02	YPKCDRAM	0.5661	109.3
5011	HLA-B*08:01	YPKCDRAM	0.4829	269.1
5015	HLA-B*51:01	DRAMPNMLRI	0.3313	1387.3
5016	HLA-B*51:01	RAMPNMLRI	0.2939	2079.5
5016	HLA-C*01:02	RAMPNMLRI	0.3299	1407.9
5016	HLA-C*03:04	RAMPNMLRI	0.7288	18.8
5016	HLA-C*06:02	RAMPNMLRI	0.3525	1102.9
5016	HLA-C*07:02	RAMPNMLRI	0.4101	591.3
5016	HLA-C*15:02	RAMPNMLRI	0.7616	13.2
5016	HLA-C*03:04	RAMPNMLRIM	0.5813	92.8
5016	HLA-C*15:02	RAMPNMLRIM	0.5101	200.4
5017	HLA-C*01:02	AMPNMLRIM	0.2496	3358
5018	HLA-B*07:02	MPNMLRIM	0.6153	64.2
5018	HLA-B*08:01	MPNMLRIM	0.5302	161.4
5018	HLA-B*07:02	MPNMLRIMA	0.5869	87.3
5018	HLA-B*35:01	MPNMLRIMA	0.5954	79.6
5030	HLA-B*08:01	LARKHTTCCSL	0.5457	136.3
5071	HLA-A*01:01	GTSSGDATTAY	0.3825	797.5
5072	HLA-A*01:01	TSSGDATTAY	0.4555	362.1
5073	HLA-A*01:01	SSGDATTAY	0.4509	380.4
5073	HLA-B*35:01	SSGDATTAY	0.531	159.9
5077	HLA-C*15:02	ATTAYANSV	0.4813	273.8
5078	HLA-B*35:01	TTAYANSVF	0.5431	140.3
5078	HLA-C*01:02	TTAYANSVF	0.2387	3779.5
5079	HLA-A*23:01	TTAYANSVFNI	0.5737	100.8
5079	HLA-A*24:02	TTAYANSVFNI	0.5682	106.9
5079	HLA-B*51:01	TTAYANSVFNI	0.3103	1741.7
5080	HLA-A*23:01	AYANSVFNI	0.7058	24.1
5080	HLA-A*24:02	AYANSVFNI	0.7086	23.4
5081	HLA-B*51:01	YANSVFNI	0.3242	1498.2
5200	HLA-B*40:01	GPHEFCSQHTM	0.5834	90.7
5202	HLA-B*40:01	HEFCSQHTM	0.7758	11.3
5202	HLA-B*44:03	HEFCSQHTM	0.5325	157.4
5202	HLA-B*40:01	HEFCSQHTML	0.733	18
5204	HLA-C*15:02	FCSQHTMLV	0.4491	387.9
5205	HLA-A*11:01	CSQHTMLVK	0.6355	51.6
5220	HLA-B*51:01	YLPYPDPSRI	0.4531	371.5
5221	HLA-B*51:01	LPYPDPSRI	0.6441	47
5221	HLA-B*07:02	LPYPDPSRIL	0.5787	95.4
5221	HLA-B*51:01	LPYPDPSRIL	0.3927	713.7
5227	HLA-C*06:02	SRILGAGCF	0.3809	810.8
5227	HLA-C*07:02	SRILGAGCF	0.4424	417.2
5227	HLA-C*06:02	SRILGAGCFV	0.3232	1514.8
5251	HLA-A*01:01	FVSLAIDAY	0.4149	561.5

5251	HLA-B*35:01	FVSLAIDAY	0.7703	12
5453	HLA-A*03:01	RLKLFAAETLK	0.6557	41.5
5454	HLA-A*03:01	LKLFAAETLK	0.6836	30.7
5455	HLA-A*03:01	KLFAAETLK	0.7776	11.1
5455	HLA-A*11:01	KLFAAETLK	0.7044	24.5
5455	HLA-A*03:01	KLFAAETLKA	0.5517	127.8
5603	HLA-A*03:01	TLQGPPGTGK	0.5476	133.5
5610	HLA-B*08:01	TGKSHFAI	0.5222	175.9
5612	HLA-C*15:02	KSHFAIGLAL	0.4933	240.5
5613	HLA-C*06:02	SHFAIGLAL	0.3431	1220.8
5613	HLA-C*07:02	SHFAIGLAL	0.4791	280.5
5713	HLA-C*06:02	ARLRAKHYV	0.3448	1198.3
5714	HLA-B*08:01	RLRAKHYV	0.5452	137.2
5714	HLA-B*08:01	RLRAKHYVYI	0.5336	155.4
5715	HLA-C*06:02	LRAKHYVYI	0.6293	55.2
5715	HLA-C*07:02	LRAKHYVYI	0.5755	98.8
5720	HLA-C*03:04	YVYIGDPAQL	0.577	97.2
5721	HLA-A*23:01	VYIGDPAQL	0.5613	115.2
5721	HLA-A*24:02	VYIGDPAQL	0.5115	197.5
5721	HLA-C*07:02	VYIGDPAQL	0.4515	377.7
5729	HLA-B*07:02	LPAPRTLL	0.6716	34.9
5729	HLA-B*08:01	LPAPRTLL	0.504	214.1
5729	HLA-B*51:01	LPAPRTLL	0.2717	2645.2
5731	HLA-B*07:02	APRTLLTKGTL	0.7391	16.8
5733	HLA-C*15:02	RTLLTKGTL	0.4932	240.6
5734	HLA-B*08:01	TLLTKGTL	0.5086	203.8
5770	HLA-B*40:01	AEIVDTVSAL	0.7215	20.4
5770	HLA-B*44:03	AEIVDTVSAL	0.5527	126.5
5771	HLA-A*01:01	EIVDTVSALVY	0.466	323
5772	HLA-C*01:02	IVDTVSALV	0.2403	3715.6
5772	HLA-C*04:01	IVDTVSALV	0.2193	4662.9
5772	HLA-C*15:02	IVDTVSALV	0.5635	112.5
5772	HLA-A*01:01	IVDTVSALVY	0.7156	21.7
5772	HLA-A*01:01	IVDTVSALVYD	0.3555	1067.8
5775	HLA-A*11:01	TVSALVYDNK	0.6916	28.1
5776	HLA-A*11:01	VSALVYDNK	0.6109	67.3
6030	HLA-B*35:01	LPLQLGFST	0.508	205.1
6032	HLA-A*02:01	LQLGFSTGV	0.7264	19.3
6069	HLA-B*08:01	DQFKHLIPL	0.4879	255
6071	HLA-C*06:02	FKHLIPLMY	0.3516	1114.3
6198	HLA-B*08:01	DAIMTRCLAV	0.4991	225.7
6198	HLA-B*51:01	DAIMTRCLAV	0.2827	2348.1
6199	HLA-B*08:01	AIMTRCLAV	0.6951	27.1
6200	HLA-B*08:01	IMTRCLAV	0.5024	217.9
6324	HLA-C*06:02	CRFDTRVL	0.369	922.6

6325	HLA-C*04:01	RFDTRVLSNL	0.2853	2282.7
6343	HLA-C*01:02	SLYVNKHAF	0.2546	3183
6390	HLA-B*51:01	YVPLKSATCI	0.3273	1448
6391	HLA-B*51:01	VPLKSATCI	0.4161	554.4
6441	HLA-A*23:01	TYNLWNTF	0.6237	58.7
6441	HLA-A*24:02	TYNLWNTF	0.615	64.4
6442	HLA-A*02:01	YNLWNTFTRL	0.6965	26.7
6445	HLA-B*08:01	WNTFTRLQSL	0.4762	289.3
6446	HLA-B*08:01	NTFTRLQSL	0.615	64.5
6446	HLA-C*01:02	NTFTRLQSL	0.2387	3778.7
6446	HLA-C*06:02	NTFTRLQSL	0.3573	1047.6
6680	HLA-B*51:01	EGYAFEHIV	0.3104	1739.5
6682	HLA-B*35:01	YAFEHIVY	0.7232	20
6683	HLA-B*44:03	AFEHIVYGDF	0.4081	604.5
6838	HLA-B*08:01	IMMNVAKYTQL	0.4816	272.9
6839	HLA-B*08:01	MMNVAKYTQL	0.6668	36.8
6844	HLA-A*23:01	KYTQLCQYL	0.6106	67.6
6844	HLA-A*24:02	KYTQLCQYL	0.6145	64.8
6844	HLA-C*07:02	KYTQLCQYL	0.432	466.5
6849	HLA-C*01:02	CQYLNTLTL	0.2485	3397.2
6849	HLA-C*03:04	CQYLNTLTL	0.549	131.6
6849	HLA-C*07:02	CQYLNTLTL	0.387	759.3
6921	HLA-C*04:01	KWDLIISDM	0.2809	2393
6924	HLA-A*11:01	LIISDMYDPK	0.6289	55.4
6925	HLA-A*11:01	IISDMYDPK	0.6456	46.3
6959	HLA-B*35:01	LALGGSVAI	0.5364	150.8
6959	HLA-B*51:01	LALGGSVAI	0.3693	920
6959	HLA-C*01:02	LALGGSVAI	0.2607	2978.4
6959	HLA-C*03:04	LALGGSVAI	0.8218	6.9
6959	HLA-C*15:02	LALGGSVAI	0.464	330.3
6960	HLA-A*03:01	ALGGSVAIK	0.603	73.3
6969	HLA-A*01:01	ITEHSWNADLY	0.7095	23.2
6970	HLA-B*40:01	TEHSWNADL	0.5867	87.5
6970	HLA-B*44:03	TEHSWNADLY	0.5926	82.1

Supplementary Table 2: SARS-CoV-2 peptide epitopes defined or used in this study

Antigen	SARS-CoV-2	Code	HLA Restriction
ORF1ab	YAFEHIVY	YAF	B*35:01
	LPYPDPSRI	LPY	B*51:01
	NVIPTITQMNL	NVI	A*02:05
	FVDGVPFVV	FVD	A*02:07
	DTDFVNEFY	DTD	A*01:01
	TTDPSFLGRY	TTD	A*01:01
	KLWAQCVQL	KLW	A*02:01
N	FPRGQGVPI	FPR	B*07:02
	RIRGGDGKM	RIR	B*07:02
	SPRWYFYYL	SPR	B*07:02
	ATEGALNTPK	ATE	A*11:01
	KPRQKRTAT	KPR	B*07:02
	AQFAPSASAF	AQF	B*15:01
	MEVTPSGTWL	MEV	B*40:01
	TPSGTWLTYTY	TPS	B*35:01
	QRNAPRITF	QRN	B*27:05
S	FEYVSQPFL	FEV	B*49:01
	YLQPRTFLL	YLQ	A*02:01
	LLQYGSFCTQ	LLQ	Not defined
	IPFAMQMAY	IPF	B*35:01
	YFPLQSYGF	YFP	A*29:01
ORF3a	FTSDYYQLY	FTS	A*01:01
	YFTSDYYQLY	YFT	A*29:01
	LLYDANYFL	LLY	A*02:01

Supplementary Table 3: Peptide epitopes sequences in human coronaviruses

Antigen	SARS-CoV-2	SARS-CoV-1	HCoV-OC43	HCoV-HKU1	HCoV-229E	HCoV-NL63
ORF1ab	YAFEHIVY	YAFEHIVY	YAFEHIVY	YAFDHIVY	FNFEHVVY	FNFEHVVY
	LPYPDPSRI	LPYPDPSRI	LPYPNPSRI	LPYPDPSRI	LPYPDPSRI	LPYPDPSRI
	NVIPTITQMNL	NVIPTITQMNL	NVLPTLTQMNL	NVLPTLTQMNL	NILPTMTQLNL	NVLPTMTQLNL
	FVDGVPFVV	FVDGVPFVV	FVDGVPFVV	FVDGVPFVV	FIDGVPVVA	FIDGVPLVT
	DTDFVNEFY	DHEFVDEFY	DSTFVTEYY	DYTFVNEYY	DESFVDDFY	EESFIDDYY
	TTDPSFLGRY	TLDESFLGRY	NFDQKELLAY	GFDQQQLLAY	GFKDAVT--F	GFEKAAL--F
	KLWAQCVQL	KLWAQCVQL	KLWHYCSTL	KLWQYCSVL	KEWAYCVEM	SEWAYCVDL
N	FPRGQGVPI	FPQGQGVPI	FVEGQGVPI	FSDGQGVPI	VIPRNLVPI	VIPRNLVPI
	RIRGGDGKM	RVRGGDGKM	SFKTADGNQ	SFKTADGQQ	RFRTRKGKR	RWRMRRGQR
	SPRWYFYYL	SPRWYFYYL	LPRWYFYYL	LPRWYFYYL	SPKLHFYYL	PPKVHFYYL
	ATEGALNTPK	ATEGALNTPK	ASNQADVNTP	ANHQADTSTP	AVDGAKTEPT	AKEGAKTVNT
	KPRQKRTAT	KPRQKRTAT	KPRQKRSPN	KPRQKRTPN	KPRWKRQPN	KPRWKRVPT
	AQFAPSASAF	AQFAPSASAF	AELAPTAGAF	AELAPTPGAF	AELVPSTAAM	AELIPNQAAL
	MEVTPSGTWL	MEVTPSGTWL	DEPQKDVYEL	DSPVKDVFEL	SKESGNTVVL	TEEVGDNVQI
	TPSGTWLTY	TPSGTWLIY	QKDVYELRY	SPVKDVFEL	SGNTVVLTF	VGDNVQITY
	QRNAPRITF	QCSAPRITF	NRSGNGILK	RSGILKKTSW	---MATVKW	---MASVNW
S	FEYVSQPFL	FEYVSQPFL	YKR---NFT	FKK---NFT	FQPLLLNCL	YQPLRLTCL
	YLQPRTFLL	YLQPRTFLL	PLTPRQYLL	PLSKRQYLL	ALASYADVL	AFATFVDVL
	LLQYGSFCTQ	LLQYGSFCTQ	LVEYGSFCDN	LSEYGTFCDN	LKQYTSACKT	LKQYTSACKT
	IPFAMQMAY	IPFAMQMAY	VPFYLNVQY	IPFSLNVQY	IPFSLAIQS	IPFSLALQA
	YFPLQSYGF	YFPLQSYGF	YCGNNSCTC	YDPRSCSQK	--------KV	--------KL
ORF3a	FTSDYYQLY	FTSDYYQLY	NA	NA	NA	NA
	YFTSDYYQLY	YFTSDYYQLY	NA	NA	NA	NA
	LLYDANYFL	LLYDANYFL	NA	NA	NA	NA

Supplementary Table 4: Conservation of SARA-CoV-2 peptides by variants

**ORF1ab 6682-6689**	**Alpha**	**Beta**	**Gamma**	**Delta**	**Epsilon**	**Zeta**	**Eta**	**Theta**	**Iota**	**Kappa**
**YAFEHIVY**	**99.92**	**100.00**	**100.00**	**99.08**	**99.92**	**100.00**	**100.00**	**100.00**	**99.91**	**100.00**
**CAFEHIVY**					**0.03**					
**YAFAHIVY**									**0.01**	
**YAFEYIVY**	**0.07**			**0.92**	**0.05**				**0.08**	
**YAFEHIIY**	**0.01**									
Number of sequences	***9498***	***357***	***6209***	***327***	***9848***	***443***	***486***	***5***	***9774***	***116***

ORF1ab 5218-5526	**Alpha**	**Beta**	**Gamma**	**Delta**	**Epsilon**	**Zeta**	**Eta**	**Theta**	**Iota**	**Kappa**
**LPYPDPSRI**	**99.93**	**92.74**	**99.98**	**100.00**	**99.90**	**99.78**	**100.00**	**100.00**	**99.91**	**100.00**
**F**PYPDPSRI		7.26	0.02		0.01				0.02	
L**S**YPDPSRI	0.06				0.04	0.22			0.05	
LPY**L**DPSRI									0.01	
LPYP**N**PSRI					0.03				0.01	
LPYPDP**A**RI					0.01					
LPYPDPSR**L**	0.01				0.01					
Number of Sequences	***9505***	***358***	***6214***	***327***	***9849***	***446***	***487***	***5***	***9797***	***116***

ORF1ab 4926-4936	**Alpha**	**Beta**	**Gamma**	**Delta**	**Epsilon**	**Zeta**	**Eta**	**Theta**	**Iota**	**Kappa**
NVIPTITQMNL	99.93	100.00	100.00	100.00	100.00	100.00	100.00	100.00	99.97	100.00
N**I**IPTITQMNL	0.02									
NV**T**PTITQMNL	0.02								0.02	
NV**V**PTITQMNL	0.03								0.01	
Number of sequences	***9500***	***358***	***6212***	***327***	***9848***	***446***	***487***	***5***	***9797***	***116***

ORF1ab 4726-4734	**Alpha**	**Beta**	**Gamma**	**Delta**	**Epsilon**	**Zeta**	**Eta**	**Theta**	**Iota**	**Kappa**
**FVDGVPFVV**	99.96	100.00	99.98	100.00	99.85	100.00	100.00	100.00	100.00	100.00
**L**VDGVPFVV	0.04									
**C**VDGVPFVV					0.14					
FVDG**I**PFVV					0.01					
FVDGVPF**A**V			0.02							
Number of sequences	***9504***	***358***	***6214***	***327***	***9850***	***446***	***487***	***5***	***9796***	***116***

ORF1ab 5130-5138	**Alpha**	**Beta**	**Gamma**	**Delta**	**Epsilon**	**Zeta**	**Eta**	**Theta**	**Iota**	**Kappa**
**DTDFVNEFY**	99.69	100.00	99.49	100.00	99.62	100.00	100.00	100.00	99.74	100.00
**Y**TDFVNEFY	0.05		0.02							
D**I**DFVNEFY	0.16				0.30				0.07	
DT**Y**FVNEFY			0.03							
DT**N**FVNEFY					0.01					
DTD**L**VNEFY	0.02									
DTDF**A**NEFY									0.12	
DTDFV**D**EFY			0.02		0.01					
DTDFV**I**EFY	0.01									
DTDFVN**D**FY	0.06		0.43		0.05				0.06	
DTDFVNEF**C**			0.02							
Number of sequences	***9502***	***358***	***6214***	***327***	***9848***	***446***	***487***	***5***	***9797***	***116***

ORF1ab 1637-1646	**Alpha**	**Beta**	**Gamma**	**Delta**	**Epsilon**	**Zeta**	**Eta**	**Theta**	**Iota**	**Kappa**
**TTDPSFLGRY**	99.17	100.00	99.66	23.55	99.68	100.00	100.00	100.00	99.73	99.11
**I**TDPSFLGRY	0.14		0.02		0.05				0.02	
**A**TDPSFLGRY									0.03	
T**A**DPSFLGRY	0.02									
T**I**DPSFLGRY					0.02				0.02	
T**N**DPSFLGRY									0.01	
TT**E**PSFLGRY									0.01	

TTD**I**SFLGRY	0.02									
TTD**L**SFLGRY	0.01			76.45	0.14				0.13	
TTD**S**SFLGRY	0.63		0.32		0.08				0.04	0.89
TTD**H**SFLGRY					0.03					
TTDPSF**M**GRY	0.01									
Number of sequences	***9503***	***357***	***6214***	***327***	***9850***	***446***	***487***	***5***	***9696***	***112***

ORF1ab 3886-3894	**Alpha**	**Beta**	**Gamma**	**Delta**	**Epsilon**	**Zeta**	**Eta**	**Theta**	**Iota**	**Kappa**
**KLWAQCVQL**	99.95	100.00	99.98	100.00	99.97	99.78	98.97	100.00	99.99	100.00
K**V**WAQCVQL	0.01									
KLW**V**QCVQL							1.03		0.01	
KLWA**R**CVQL	0.01		0.02		0.03					
KLWA**E**CVQL	0.02									
KLWAQC**I**QL	0.01									
KLWAQCV**R**L						0.22				
Number of sequences	***9505***	***358***	***6214***	***327***	***9848***	***446***	***487***	***5***	***9796***	***116***
